# Porcine non-conventional B-1-like cells are a potent source of *Streptococcus suis*-binding IgM

**DOI:** 10.3389/fimmu.2024.1495359

**Published:** 2024-11-18

**Authors:** Anne-Marie Seidel, Johannes Kauffold, Martina Protschka, Christoph G. Baums, Gottfried Alber, Maria Eschke

**Affiliations:** ^1^ Institute of Immunology, Centre for Infectious Diseases, Faculty of Veterinary Medicine, Centre for Biotechnology and Biomedicine, Leipzig University, Leipzig, Germany; ^2^ Clinic for Ruminants and Swine, Faculty of Veterinary Medicine, Leipzig University, Leipzig, Germany; ^3^ Institute of Bacteriology and Mycology, Centre for Infectious Diseases, Faculty of Veterinary Medicine, Leipzig University, Leipzig, Germany

**Keywords:** *Streptococcus suis*, IgM, pig, swine, non-conventional B cells, innate B-1 cells, B-2 cells, cell culture

## Abstract

**Introduction:**

*Streptococcus suis* serotype (*cps*) 2 is an important bacterial pathogen in piglet nurseries and an emerging zoonotic agent without effective vaccines available. Immunoglobulin (Ig)M plays an essential role in host defense against *S. suis*. In mice, non-conventional B-1 cells are a major source of protective IgM against encapsulated bacterial pathogens, such as *S. pneumoniae*. Two IgM^+^CD21^−^ B-1-like cell subpopulations, distinguishable by CD11R1 expression, were described in pigs, but their properties and functions are poorly understood. This study aimed at a first characterization of the porcine early IgM B cell response against *S. suis cps* 2.

**Methods:**

We analyzed the same healthy pigs, naturally colonized by different *S. suis* serotypes, including *cps* 2, at four and eight weeks of age serologically and determined the frequency of different peripheral B cell subpopulations by flow cytometry. Furthermore, we isolated conventional IgM^+^CD21^+^ B-2 cells as well as non-conventional B-1-like cell subpopulations from peripheral blood of eight-weeks-old pigs to evaluate their potential of IgM secretion in response to innate and adaptive stimuli *in vitro*.

**Results:**

Between the fourth and eighth week of life, a characteristic increase of *S. suis cps* 2-binding serum IgM antibodies, restricting bacterial growth, was observed. Moreover, we show for the first time that the significant increase of anti-*S. suis* serum IgM is associated with a relative increase of peripheral non-conventional IgM^+^CD21^−^ B-1-like cells *in vivo*, particularly of the IgM^+^CD21^−^ CD11R1^−^ subpopulation. Noteworthy, sorted IgM^+^CD21^−^ CD11R1^−^ B-1-like cells from eight-weeks-old pigs spontaneously secreted IgM *in vitro*. In addition, both non-conventional IgM^+^CD21^−^ B cell subpopulations, in contrast to conventional IgM^+^CD21^+^ B-2 cells, produced anti-*S. suis* IgM upon toll-like receptor (TLR) stimulation underlining their innate-like characteristics. We furthermore observed that both B-1-like subpopulations secrete *S. suis cps* 2-binding IgM upon stimulation with T cell-associated factors with highest amounts in IgM^+^CD21^−^CD11R1^−^ B-1-like cells even exceeding anti-*S. suis* IgM levels produced by B-2 cells.

**Conclusion:**

Porcine non-conventional B-1-like cells are a potent source of *S. suis*-binding IgM indicating a role in immunity during a critical phase of piglet rearing.

## 1 Introduction

*Streptococcus suis* is an encapsulated, Gram-positive, zoonotic bacterial pathogen (1). As a natural colonizer, *S. suis* can be ubiquitously found on tonsils and mucosal surfaces of healthy pigs (2). However, invasive *S. suis* infection causes life-threatening disease, including meningitis and septicemia in pigs and also in humans (3–5). In piglet nurseries, *S. suis* is one of the most important pathogens and is associated with high economic losses (5, 6). Pigs at the age of five to ten weeks are predominantly affected (7). Due to the lack of effective vaccines, *S. suis* is one of the main reasons for the extensive use of antimicrobials in pigs (6). Based on differences in capsular polysaccharides (*cps*), *S. suis* is classified into 29 serotypes (8, 9). However, this number does not account for several novel capsular types that have recently been recognized using molecular methods (9). While the serotype distribution of *S. suis* varies locally, *S. suis* serotype 2 (*cps* 2) is the most relevant serotype in pigs and humans worldwide (10).

Bacteremia is considered a crucial step in the pathogenesis of invasive *S. suis* infections and the bacterial polysaccharide capsule serves as a virulence factor providing protection against opsonophagocytosis in the blood (11–14). Previous studies demonstrated a role of IgM in host defense (15, 16) (IgM-complement-oxidative burst-axis), which is particularly important in the absence of or at low levels of *S. suis*-binding IgG (16). *S. suis* secretes an immunoglobulin M-degrading enzyme (Ide*_Ssuis_*), which serves as a complement evasion mechanism (17). Murine monoclonal IgM antibodies directed against *S. suis cps* 2 were shown to mediate the killing of *S. suis* serotype 2 *in vitro* by opsonization triggering phagocytosis and/or agglutination. This correlated with protection after passive immunization in the mouse model (18).

Conventional B cells, also termed B-2 cells, are an essential component of the adaptive immune system providing antibody-mediated protection against infections. Conventional B-2 cells recognize non-self protein antigens and secrete specific IgM for a short time after activation before usually switching to the production of IgG (19, 20). In addition, B-1 cells with innate functions have been described as a distinct non-conventional B cell lineage in mice and humans. B-1 cells are the main producers of natural IgM antibodies with polyspecific reactivity against self and microbial antigens. Antibodies constitutively secreted by B-1 cells assist in the disposition of cellular debris and provide a first line defense against a variety of microbial pathogens (20). In addition to providing a steady-state level of natural IgM, B-1 cells have also been shown to actively respond to infections with induced IgM production (21). Activation of B-1 cells is induced by repetitive sequences, like capsule polysaccharides, as demonstrated in human immunization studies with pneumococcal polysaccharide (22). In mice, non-conventional B-1 cells have been shown to release large amounts of protective capsule-specific IgM following recognition of polysaccharides of bacterial pathogens, such as *S. pneumoniae* (23). Although B-1-like cells have been described in pigs (24), little is known about their properties and function.

This study aimed at a first characterization of the early porcine IgM B cell response against *S. suis cps* 2 and included the analysis of non-conventional B-1-like subpopulations in comparison to conventional B-2 cells.

In pigs between four and eight weeks of age we observed a characteristic increase of *S. suis cps* 2-binding serum IgM, which restricts survival of streptococci in porcine blood *in vitro.* Moreover, we show that this IgM increase is associated with the relative increase of IgM^+^CD21^−^ B-1-like cells in peripheral blood *in vivo*. CD21^−^CD11R1^−^ B-1-like cells of 8-weeks-old pigs, capable of constitutive IgM production, are the major source of anti-*S. suis* IgM *in vitro* in response to toll-like receptor (TLR) stimulation and T cell-associated signals. Our study characterizes the early porcine IgM B cell response and demonstrates its importance in a critical phase of piglet rearing.

## 2 Material and methods

### 2.1 *S. suis* serotype (*cps*) 2 strain 10 growth conditions

*S. suis* serotype (*cps*) 2 strain 10 is a virulent strain of sequence type 1 that has previously been used for pathogenesis studies by different groups (25, 26), kindly provided by H. Smith (DLO-Lelystad, The Netherlands) (27). Bacteria were grown overnight at 37 °C in Bacto™ Todd-Hewitt-Broth (THB) (BD, Heidelberg, Germany) or on Columbia agar plates with 6% sheep blood (Thermo Oxoid, Schwerte, Germany). *S. suis* glycerol stocks were prepared at the exponential growth phase (optical densitiy (OD)_600 nm_ = 0.5) and stored at −80 °C in 15% glycerol as single-use aliquots.

### 2.2 Expression and purification of recombinant Ide*_Ssuis_* _homologue

The recombinant Ide*_Ssuis_* _homologue (rIde*_Ssuis_* _h) is a truncated variant of the enzyme including the conserved IgM protease domain. Functionally it is equivalent to wild-type full-length Ide*_Ssuis_* (25). Expression was induced in *Escherichia coli* BL21 (DE3), carrying the pET*ide*Ssuis_homologue plasmid, by the addition of 1 mM isopropyl-β-D-thiogalactopyranoside (IPTG) during the exponential growth phase and the protein was subsequently purified using Ni-affinity chromatography as described previously (16, 25).

### 2.3 Pigs, blood sample collection, and isolation of porcine peripheral blood mononuclear cells

Blood collection from conventional healthy post-weaning pigs (boar: Hypor Libra × sow: PIC408) naturally colonized by different *S. suis* serotypes, including *cps* 2, was approved under the permit number 81-02.04.40.2023.VG034 by North Rhine-Westphalia State Agency for Nature, Environment and Consumer Protection (*Landesamt für Natur, Umwelt und Verbraucherschutz Nordrhein-Westfalen*). Whole blood and serum samples from the same pigs at four and eight weeks were collected via the *Vena cava cranialis* into heparinized tubes (Monovette^®^, Sarstedt, Nümbrecht, Germany) or serum tubes (Monovette^®^, Sarstedt), respectively. Whole blood samples were centrifuged at 400×g for 10 min, and the plasma was removed. For isolation of peripheral blood mononuclear cells (PBMC), blood was diluted with an equal volume of phosphate buffered saline (PBS), layered onto Pancoll^®^ Separating Solution (density 1.077 g/mL, PAN-Biotech, Aidenbach, Germany) and centrifuged (900×g, 35 min at room temperature (RT), minimal acceleration and deceleration). Mononuclear cells from the interphase were collected and washed with medium (IMDM with L-Glutamine, 25 mM HEPES and 3.024 g/L NaHCO_3_; PAN-Biotech) supplemented with 10% heat-inactivated fetal bovine serum (FBS, PAN-Biotech) (500×g, 12 min, RT), followed by another washing step with PBS (400×g, 10 min, RT). If required, lysis of remaining erythrocytes was performed by incubating cells at RT in 1-2 mL of lysis buffer (150 mM NH_4_Cl, 8 mM KHCO_3_, 2 mM EDTA in PBS, pH 7.0) for 2-3 min. Medium containing 10% FBS was added to stop the reaction. Following two washing steps (400×g, 6 min, RT), the cell number was determined using a Neubauer chamber (Laboroptik Lancing, UK), excluding dead cells via trypan blue staining (Merck-Sigma, Darmstadt, Germany). PBMC were used immediately for phenotyping of B cell subpopulations by flow cytometric analysis or cryo-preserved in liquid nitrogen until B cell sorting [freezing medium: 50% FBS, 40% IMDM, 10% dimethyl sulfoxide (DMSO, Merck-Sigma)].

### 2.4 Enzyme-linked immunosorbent assay (ELISA)

Detection of anti-*S. suis* IgM and IgG in serum samples was performed as described previously (17). Briefly, Nunc MaxiSorp™ flat bottom plates (ThermoFisher, Darmstadt, Germany) were coated overnight (O/N) at 4°C with 1×10^7^ colony-forming units (CFU) of inactivated (0.2% paraformaldehyde O/N) *S. suis cps* 2 strain 10 per well in PBS. All incubations described below were followed by washing steps with PBST (PBS, 0.05% v/v Tween20, Carl Roth). After blocking with a combination of 0.5% w/v BSA and 0.1% v/v gelatine (Carl Roth) in PBS for 1 hour at 37°C, four-step serial dilutions of serum samples were added in duplicates and incubated for 1.5 hours at 37°C. Anti-*S. suis* IgG was detected using peroxidase-conjugated goat anti-swine IgG (50 ng/mL, Bethyl Laboratories, Montgomery, Texas), while IgM was detected using peroxidase-conjugated goat anti-swine IgM (100 ng/mL, Bethyl Laboratories). 3,3’,5,5’-Tetramethylbenzidine substrate (TMB, KPL, medac, Wedel, Germany) was added for colorimetric development. After 15 min (assay-optimized), the reaction was stopped with 1 M phosphoric acid (Carl Roth). The OD at 450 nm was measured using a Spectra-Max 340 ELISA reader with SoftMax Pro software v5.0 (Molecular devices, Munich, Germany). Blank-reduced ODs were converted to ELISA units using a standard serum from a pig prime-booster-vaccinated with *S. suis cps* 2 strain 10 as internal reference defined to include 100 ELISA units. The standard serum as well as the colostrum-deprived serum serving as negative control have been used previously (28).

Anti-*S. suis* IgM was determined in supernatants of stimulated B cell subpopulations (see sections 2.7 and 2.8) as described above for serum samples. Supernatants were used undiluted. Blank-reduced ODs were reported and compared.

For determination of total IgM in serum and supernatants of medium-incubated/stimulated B cell subpopulations, ELISA plates were coated with 2.4 µg/mL of monoclonal anti-porcine IgM (clone: K521C3) Biorad, Feldkirchen, Germany) in PBS. Supernatants of cells incubated in medium without stimuli were diluted 1:2, while serial dilutions of sera (starting from 1:10,000) as well as of supernatants from stimulated cells (CD40L plus IL-21 starting from 1:100; Pam3Cys-SK4 starting from 1:10) were used. Detection was performed as described above. Swine IgM whole molecule serum (Rockland Immunochemicals, Limerick, Pottstown) was used as a standard and total IgM concentrations were calculated based on the standard curve.

### 2.5 *S. suis cps* 2 whole blood bacterial survival assay

Survival of *S. suis cps* 2 strain 10 in porcine blood *ex vivo* was determined following the protocol of Seele et al., 2013 (25). Briefly, 500 μL of heparinized blood was mixed with 1×10^6^ CFU of exponentially grown bacteria. CFU were determined by serial dilutions at time zero and after incubation of the samples in a rotator for 2 hours at 37°C. The survival factor was defined as the ratio of CFU at 120 min to CFU at time zero. Where indicated, 10 µg of rIdeSsuis_h was added to evaluate the impact of IgM on bacterial survival in blood of pigs at four and eight weeks of age.

### 2.6 Phenotyping of B cell subpopulations by flow cytometric analysis

The frequency of conventional and non-conventional B cell subpopulations in peripheral blood was determined in the same pigs at four and eight weeks of age by flow cytometry. A fixable viability dye (eFluor 506, Thermo Fisher Scientific, Carlsbad, USA) was used according to the manufacturer’s protocol to allow for discrimination between viable and dead cells. To prevent binding via Fc receptors, cells were incubated with heat-inactivated normal serum derived from pig (30% in PBS). All surface staining steps described below were performed for 20 min on ice in the dark and separated by washing steps with FACS buffer (PBS, 3% FBS, 0.1% sodium azide (Merck-Sigma); 500×g, 3 min, 4°C). Detailed information regarding the applied primary and secondary antibodies for flow cytometric staining is provided in **Table 1**. Initially, cells were incubated with anti-porcine CD11R1, anti-porcine IgG, and anti-porcine IgM. Detection of CD11R1 was achieved using a fluorescently labeled anti-mouse IgG1 secondary antibody (**Table 1**). In the final surface staining step, cells were incubated with a mixture containing the remaining primary antibodies (anti-CD21, anti-CD3) as well as secondary antibodies for detection of IgM and IgG (**Table 1**). For intracellular staining of the pan-B-cell marker CD79a (**Table 1**), cells were permeabilized using the FoxP3/Transcription Factor Staining Buffer Set (Thermo Fisher Scientific, Carlsbad, USA) according to the manufacturer’s protocol. After permeabilization, an additional blocking step with 30% heat-inactivated porcine normal serum was done and cells were incubated with the staining antibody for 30 min at RT. Following acquisition with a BD LSR Fortessa™ flow cytometer (Becton Dickinson (BD), Heidelberg, Germany) equipped with BD FACS Diva ™ software version 6.1.3 (BD), data were analyzed using the FlowJo™10.10 software (BD). After exclusion of doublets and dead cells, PBMC were gated on CD79a^+^IgM^+^ B lymphocytes and the frequency of non-conventional IgM^+^CD21^-^CD11R1^+^ and IgM^+^CD21^-^CD11R1^-^ B-1-like subpopulations as well as of conventional IgM^+^CD21^+^ B-2 cells was analyzed. The frequency of IgG^+^ B cells was analyzed for comparison. Adequate gating was performed according to Fluorescence Minus One (FMO) controls included in the experiments.

**Table 1:** Antibodies and reagents used for flow cytometric analysis and fluorescent activated cell sorting.

Name/Specificity	Clone	Isotype	Labeling strategy	Fluorochrome	Source
CD11R1*	Mil4	mouse IgG1	secondary antibody^a^	Brilliant Violet (BV)421	Bio-Rad
IgM	polyclonal	goat Ig	secondary antibody^b^	FITC	Bethyl
IgG	MT424	mouse IgG2a	Biotin-streptavidin^c^	BV605	Mabtech
CD3ϵ	BB23-8E6-8C8	mouse IgG2a	directly conjugated	PE-Cy7	BD Biosciences
CD21	REA940	human IgG1	directly conjugated	PE, APC	Miltenyi
CD79a**	HM47	mouse IgG1	directly conjugated	PE	BioLegend

a rat anti-mouse IgG1 (clone RMG1-1), Brilliant Violet 421, BioLegend.

b donkey anti-goat IgG (polyclonal), FITC, BioLegend.

c Streptavidin-BV605, BioLegend.

*probably the porcine homolog of CD11b (46).

**intracellular detection.

### 2.7 Sorting of B cell subpopulations

The different conventional and non-conventional B cell subpopulations were isolated from PBMC of the same individual pigs at four and eight weeks of age by fluorescence-activated cell sorting (FACS). Viability dye and surface staining (**Table 1**) were performed as described above, except for that FACS buffer was replaced by PBS, 3% FCS (without sodium azide) in the protocol. Stained cells were passed through a 30 µm filter (Sysmex Germany GmbH, Norderstedt, Germany) and immediately sorted through a 85 μm nozzle using a BD FACSAria™ III Cell Sorter (BD, Heidelberg, Germany) equipped with BD FACS Diva ™ software version 6.1.3. Four B cell subpopulations were collected: IgG^+^, conventional IgM^+^CD21^+^ B-2 as well as non-conventional IgM^+^CD21^-^CD11R1^+^ and IgM^+^CD21^-^CD11R1^-^ B-1-like cells. Purity was determined by post-sort re-analysis using FlowJo™ 10.10 software (BD).

### 2.8 Stimulation of sorted B cell subpopulations

The four sorted B cell subpopulations were seeded at a density of 5×10^4^ per well in culture medium (IMDM with L-Glutamine, 25 mM HEPES and 3.024 g/L NaHCO_3_; PAN-Biotech) supplemented with 10% heat-inactivated FBS, 100 U/mL penicillin, and 100 μg/mL streptomycin (both purchased from PAA Laboratories, Cölbe, Germany) containing 50 ng/µL porcine IL-2 (BioLegend, San Diego, United States) into a round-bottom 96-well cell culture plate (TPP, Trasadingen, Switzerland). To evaluate the IgM response of sorted B cell subpopulations to Toll-like-receptor (TLR) stimulation and T cell-associated signals, cells were stimulated with 10 µg/mL Pam3Cys-SK4 (InvivoGen, Toulouse, France) or 1 µg/mL human CD40L plus 50 ng/mL human IL-21 (both: BioLegend), respectively. To promote cell sedimentation, plates were centrifuged at 400×g for 2 min before start of culture (29). B cell subpopulations were incubated for 3 days at 37°C and 5% CO_2_. The viability of the different B cell subpopulations was determined using a viability dye by flow cytometry after culture. Importantly, the viability of the different B cell subpopulations was comparable for the individual stimulation conditions allowing for a comparison of IgM secretion.

### 2.9 Statistical analysis

Statistical analysis was performed using GraphPad Prism 10.2.0 (GraphPad Software Inc., San Diego, USA). The Shapiro-Wilk test was applied to test for normal distribution. Normally distributed data are presented with means. Nonparametric data are shown with medians. Depending on normal distribution, the paired t-test or the Wilcoxon matched-pairs signed-rank test (both: two-tailed) were used for statistical analysis. Significance of stimulation-induced effects was calculated for each subpopulation by direct comparison of medium versus the CD40L plus IL-21 or Pam3Cys-SK4-stimulated equivalent. A Pearson correlation analysis was conducted between *S. suis*-binding IgM and total IgM. The level of confidence for significance is shown in figure legends.

## 3 Results

### 3.1 *S. suis cps* 2-binding serum IgM increases in pigs between four and eight weeks of age and restricts survival of streptococci

Since invasive infections of *S. suis* mainly affects pigs at the age of five to ten weeks, understanding changes in the immune status after the forth week of life is of great importance. Here, in healthy, weaned pigs naturally colonized with different serotypes of *S. suis*, including *cps* 2, we analyzed anti-*S. suis cps* 2 antibody levels during this critical period.

**Figure 1:**
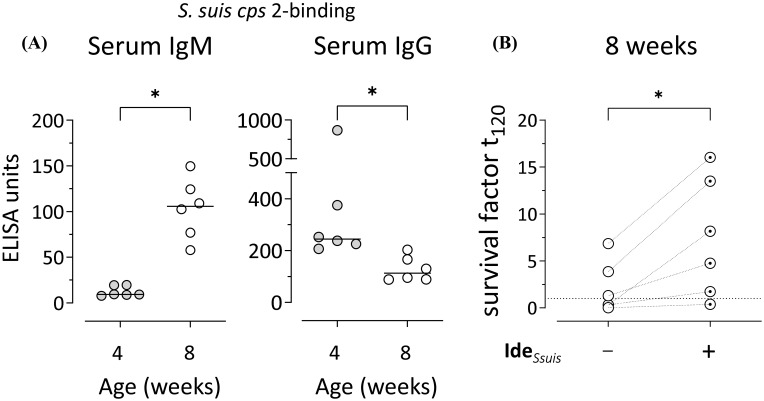
*S. suis*-binding serum IgM increases between four and eight weeks of age and restricts bacterial survival *in vitro*. **(A)** *S. suis cps* 2-binding serum IgM and IgG were analyzed in the same pigs at four and eight weeks of age by ELISA. Plates were coated with whole inactivated bacteria of *S. suis*. A hyperimmune serum pool defined to include 100 ELISA units served as internal reference. Data of one out of two independent experiments are shown and presented with group medians. Statistical analysis was performed using Wilcoxon matched-pairs signed-rank test. (two-tailed, *p<0.05). **(B)** Survival of *S. suis cps* 2 strain 10 was assessed in the blood drawn from eight-weeks-old pigs in the presence/absence of the specific IgM protease Ide*_Ssuis_*. The survival factor represents the ratio of CFU at 120 min to CFU at time zero. Survival factors above one (dotted line) indicate proliferation of *S. suis*. Data of one out of two independent experiments are shown. Each dot represents one individual pig. Statistical analysis was performed using paired t-test. (two-tailed, *p<0.05).

While *S. suis cps* 2-binding IgM was low in 4-weeks-old pigs, it significantly increased until eight weeks of age (**Figure 1A**). In contrast, anti-*S. suis cps* 2 IgG decreased over the same period (**Figure 1A**), indicating stable IgM production without indication of isotype switching to IgG between four and eight weeks. These findings are in line with previous reports showing a similar course of anti-*S. suis* IgM and IgG levels in porcine blood for other serotypes (*cps* 1, 7, 14/1) (30, 31). Using an established *in vitro* whole blood bacterial survival assay (30), comparable survival of *S. suis cps* 2 was observed in the blood of the same pigs at four and eight weeks of age (**Supplementary Figure 1A**). To elucidate the role of IgM in control of *S. suis cps* 2 in porcine blood *in vitro*, Ide*_Ssuis_*, a highly specific protease cleaving porcine IgM, but not IgG (25), was added in the whole blood bacterial survival assay. As expected from the low serum IgM levels (**Figure 1A**), incubation with the IgM protease did not alter the survival of *S. suis cps* 2 in blood of 4-weeks-old pigs (**Supplementary Figure 1B**). In contrast, presence of Ide*_Ssuis_* significantly increased the survival of *cps* 2 in blood of the same pigs at the age of eight weeks (**Figure 1B**). In blood samples of five out of six 8-week-old pigs, even an increase in the survival factors above values of one was observed after addition of the IgM protease, indicating proliferation of *S. suis cps* 2 upon IgM cleavage (**Figure 1B**). Comparable survival of *S. suis cps* 2 in the blood of the same pigs at four and eight weeks of age (**Supplementary Figure 1A**) likely can be attributed to higher maternal anti-*S. suis* IgG levels in the former and higher anti-*S. suis* IgM levels in the latter (**Figure 1A**). In serum from 8-weeks-old pigs, levels of anti-*S. suis* IgM and total IgM correlated (**Supplementary Figure 2**).

Taken together, our data indicate that the increase of anti-*S. suis cps* 2 IgM in porcine serum after the fourth week of life contributes to the restriction of survival and proliferation of the bacterial pathogen.

### 3.2 Relative increase of non-conventional B-1-like subpopulations in the peripheral blood of pigs between four and eight weeks of age

**Figure 2:**
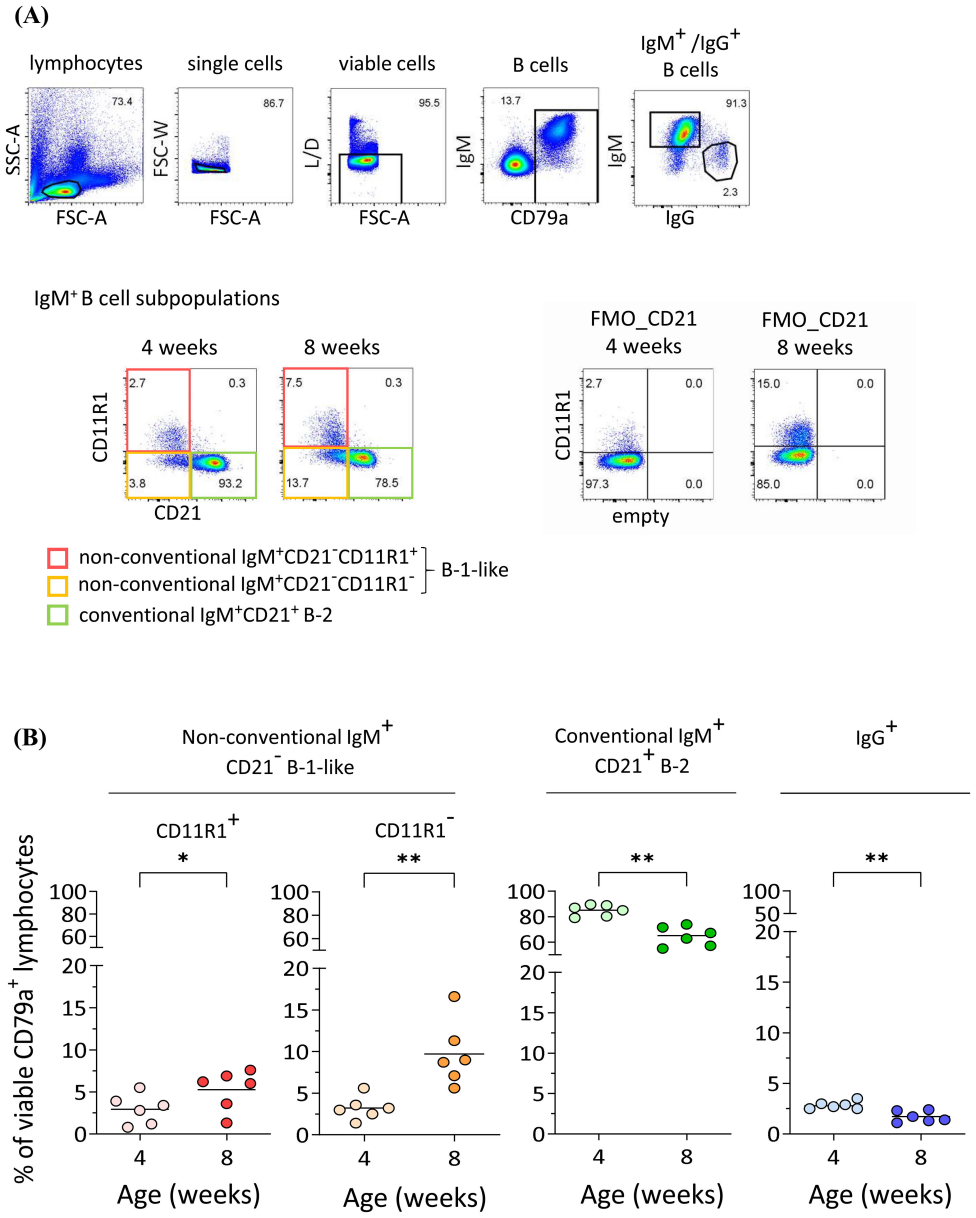
Increase of non-conventional IgM^+^CD21^-^ B-1-like cell populations in peripheral blood of pigs between four and eight weeks of age. **(A)** The frequency of conventional and non-conventional IgM^+^ B cell subpopulations in peripheral blood in the same pigs at four and eight weeks of age was determined by flow cytometry. Representative pseudocolor plots of PBMC gated on CD79a^+^IgM^+^ B lymphocytes after exclusion of doublets and dead cells show an increase in the frequency of both, non-conventional IgM^+^CD21^-^CD11R1^+^ and IgM^+^CD21^-^CD11R1^-^ B-1-like subpopulations, and a relative decrease in conventional IgM^+^CD21^+^ B-2 cells. The frequency of IgG^+^ B cells was analyzed for comparison. FMO: fluorescence minus one control. **(B)** Quantification of indicated B cell subpopulations in peripheral blood of the same pigs at four and eight weeks of age. Data of one out of two independent experiments are shown. Each dot represents one individual pig, the horizontal bars indicate mean values. Paired t-test was performed for statistical analysis (two-tailed, *p<0.05, **p < 0.01).

Next we were interested in the cellular source of anti-*S. suis* IgM. In mice, non-conventional B-1 cells produce protective IgM antibodies against *S. pneumoniae* (23). Here, we applied a multicolor flow cytometric staining protocol (24) to identify conventional and non-conventional B cell subpopulations in the peripheral blood of the same pigs at four and eight weeks of age (**Figure 2A**). PBMC from the same pigs whose serum IgM and IgG data are shown in **Figure 1** were used. After excluding doublets and dead cells in flow cytometric analysis, we identified B cells using CD79a as a marker and analyzed four B cell subpopulations: non-conventional IgM^+^CD21^−^CD11R1^+^ and IgM^+^CD21^−^CD11R1^−^ B-1-like cells as well as conventional IgM^+^CD21^+^, and IgG^+^ B-2 cells. Our analysis revealed an increase in the frequency of non-conventional IgM^+^CD21^−^CD11R1^+^ (mean 1.8-fold) and IgM^+^CD21^−^CD11R1^−^ (mean 3.0-fold) B-1-like cells in porcine blood between four and eight weeks of age, while a relative decrease of conventional IgM^+^CD21^+^ B-2 cells was observed (**Figure 2**). The relative proportion of IgG^+^ B-2 cells also decreased in this period (**Figure 2**). Thus, the significant increase of *S. suis cps* 2-binding IgM in peripheral blood of four to eight weeks old pigs (**Figure 1**) is associated with an increase of the frequency of non-conventional IgM^+^CD21^−^ B-1-like cells, most pronounced for the CD11R1^−^ subpopulation (**Figure 2B**).

### 3.3 Porcine IgM^+^CD21^−^CD11R1^−^ B-1-like cells constitutively secrete IgM and are highly reactive to toll-like receptor (TLR) stimulation and T cell-associated signals

**Figure 3:**
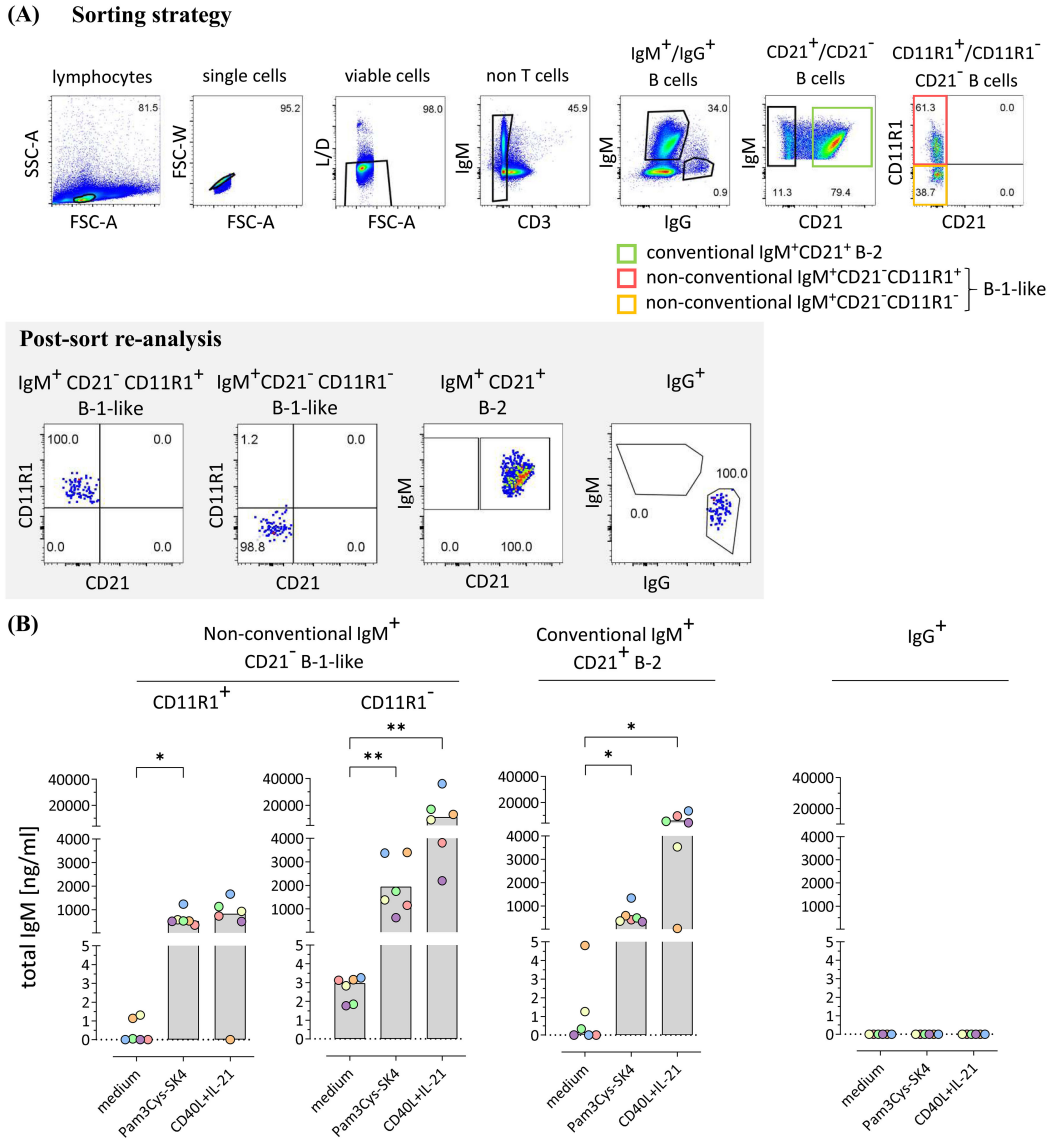
Porcine CD21^-^CD11R1^-^ B-1-like cells are capable of constitutive IgM production and secrete high amounts of IgM in response to toll-like receptor (TLR) stimulation and T cell-associated signals **(A)** The strategy for isolation of different B cell subpopulations from peripheral blood mononuclear cells of 8-weeks-old pigs by fluorescence-activated cell sorting is shown. After gating on single viable CD3^-^ lymphocytes, conventional IgG^+^ as well as IgM^+^CD21^+^ B-2 and non-conventional IgM^+^CD21^−^ B-1-like B cell subpopulations were selected. The latter were further divided according to CD11R1 expression to obtain four different fractions: (1) IgM^+^CD21^−^ CD11R1^+^, (2) IgM^+^CD21^−^ CD11R1^−^, (3) IgM^+^CD21^+^, and (4) IgG^+^. High purity (>98%) of the isolated populations was confirmed by re-analysis. Representative data of one pig are shown. Numbers in gates depict percentages. **(B)** The sorted conventional and non-conventional B cell subpopulations were incubated for three days in medium or stimulated with either the TLR ligand Pam3Cys-SK4 or the T cell-associated factors CD40L and IL-21 in the presence of IL-2. Secretion of total IgM was analyzed by ELISA. Depending on normal distribution, data sets are presented with the mean (CD11R1^+^) or the median. Significance of stimulation-induced effects was calculated for each subpopulation by direct comparison of medium versus the Pam3Cys-SK4 or CD40L plus IL-21 stimulated equivalent using the paired t test or the Wilcoxon matched-pairs signed-rank test, respectively (both: two-tailed, *p<0.05, **p<0.01).

To assess the potential of IgM secretion by the individual IgM^+^ B cell subpopulations, conventional IgM^+^CD21^+^ B-2 cells as well as non-conventional IgM^+^CD21^−^CD11R1^+^ and IgM^+^CD21^−^CD11R1^−^ B-1-like cell subpopulations were isolated by fluorescence-activated cell sorting from PBMC of 8-weeks-old pigs with high purity (**Figure 3A**). IgG^+^ B cells were isolated for control (**Figure 3A**). The sorted B cell subpopulations were incubated in medium for three days or stimulated with either the Toll-like receptor (TLR) 1/2 ligand Pam3Cys-SK4 to analyze innate B cell activation or, to characterize adaptive T cell dependent B cell activation, with the T cell-associated factors CD40L and IL-21 (32). Responsiveness to TLR stimulation is a well-known feature of B-1 cells (24, 33, 34) and murine B-1 cells have also been shown to respond to T cell signals *in vitro* (35). Of note, analysis of total IgM in supernatants of purified B cell subpopulations by ELISA revealed spontaneous IgM secretion under medium condition only by non-conventional IgM^+^CD21^−^CD11R1^−^ B-1-like cells (**Figure 3B**). Moreover, IgM^+^CD21^−^CD11R1^−^ B-1-like cells responded to TLR stimulation with highest IgM production (**Figure 3B**). Conventional IgM^+^CD21^+^ B-2 and non-conventional IgM^+^CD21^−^CD11R^+^ B-1-like cells also secreted IgM following TLR stimulation, albeit to a slightly lesser extent (**Figure 3B**). Similar to conventional IgM^+^CD21^+^ B-2 cells and in contrast to the IgM^+^CD21^−^CD11R1^+^ B-1-like subpopulation, IgM^+^CD21^−^CD11R^−^ B-1-like cells secreted high amounts of IgM in response to the T cell-associated signals CD40L and IL-21 (**Figure 3B**). As expected IgM was not detectable in supernatants from sorted IgG^+^ B cells (**Figure 3B**). Thus, IgM^+^CD21^−^CD11R1^−^ B-1-like cells are not only a potent source of induced IgM under innate and adaptive B cell stimulation conditions, but also constitutively secrete IgM conceivable with pronounced innate features of this B cell subpopulation.

### 3.4 Non-conventional IgM^+^CD21^−^ B-1-like cells secrete *S. suis*-binding IgM upon TLR stimulation in contrast to conventional IgM^+^CD21^+^ B-2 cells, with highest levels in the CD11R1^−^ subpopulation

**Figure 4:**
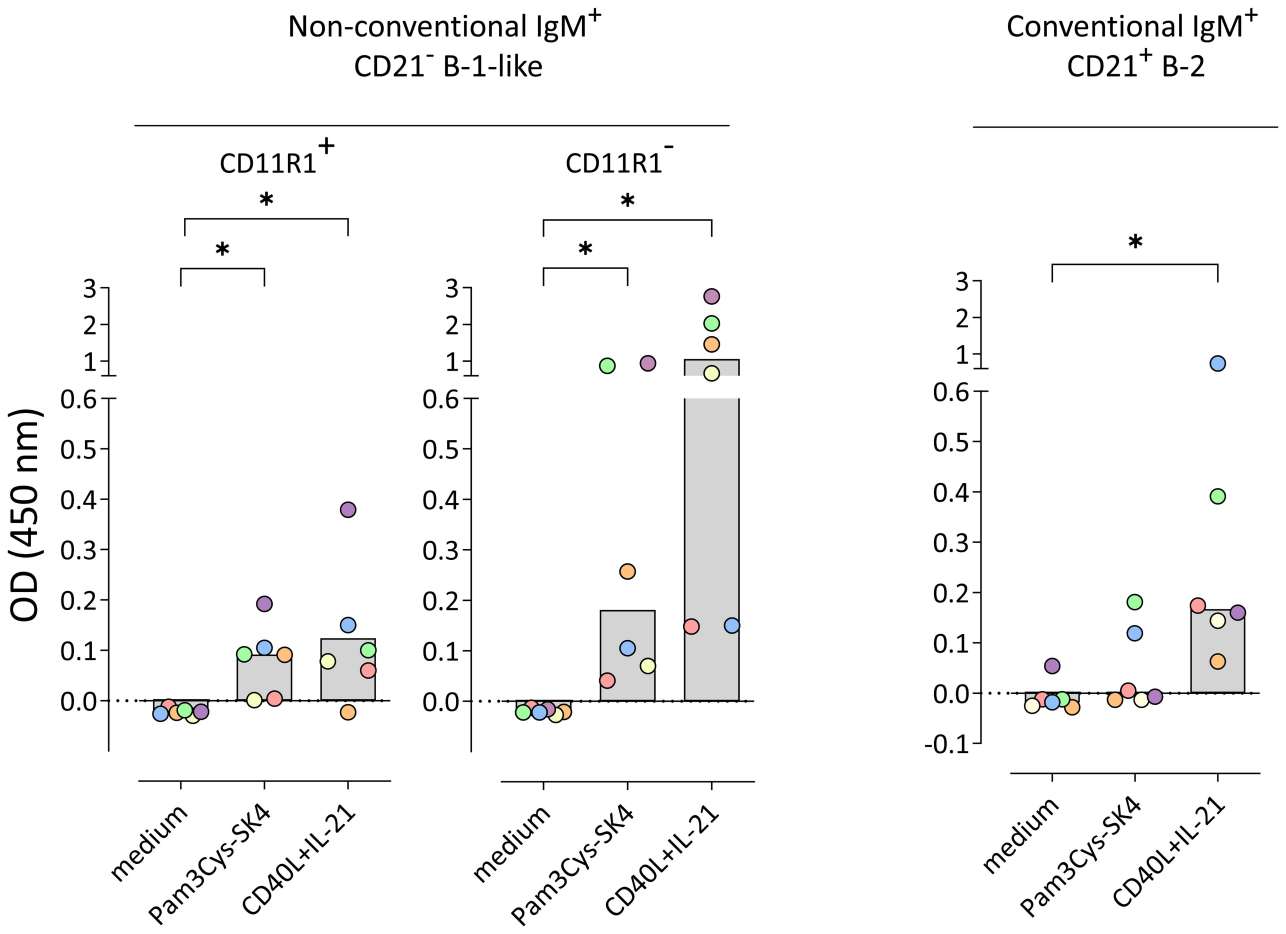
Non-conventional IgM^+^CD21^−^ B-1-like cells but not conventional IgM^+^CD21^+^ B-2 cells isolated from peripheral blood of 8-weeks-old pigs secrete *S. suis cps* 2-binding IgM in response to toll-like receptor (TLR) stimulation, with highest levels in the CD11R1^−^ subpopulation. Conventional and non-conventional IgM^+^ B cell subpopulations were sorted in four independent experiments from a total of six 8-week-old pigs. Isolated B cell subpopulations were incubated for three days in medium or stimulated with either the TLR ligand Pam3Cys-SK4 or the T cell-associated factors CD40L and IL-21 in the presence of IL-2. The amount of *S. suis cps* 2-binding IgM in supernatants was determined using whole inactivated bacteria as coating antigen. Blank-reduced optical densities (OD) are shown. Each colored dot represents one individual pig. Depending on normal distribution, data sets are presented with the mean or the median. Significance of stimulation-induced effects was calculated for each subpopulation by direct comparison of medium versus the Pam3Cys-SK4 or CD40L plus IL-21 stimulated equivalent using the paired t test or the Wilcoxon matched-pairs signed-rank test, respectively (both: two-tailed, *p<0.05).

Next, we investigated whether the constitutively produced IgM detected in the supernatant of IgM^+^CD21^−^CD11R1^−^ B-1-like cells and the stimulation-induced IgM detected in supernatants of conventional and non-conventional B cell subpopulations bind to *S. suis cps* 2. For that purpose, ELISA plates were coated with whole inactivated bacteria and supernatants analyzed for total IgM (**Figure 3B**) were additionally analyzed for binding to *S. suis cps* 2. As shown in **Figure 4**, constitutively produced IgM binding to *S. suis cps* 2 was not detectable (**Figure 4**) likely due to limited amounts of total IgM as compared with the >700-fold higher stimulation-dependent levels (see **Figure 3B** for difference between constitutive and induced total IgM secretion). Noteworthy, in contrast to conventional IgM^+^CD21^+^ B-2 cells, both non-conventional IgM^+^CD21^−^ B-1-like cell subpopulations secreted anti-*S. suis* IgM following stimulation with the TLR 1/2 ligand Pam3Cys-SK4 (**Figure 4**). Furthermore, upon stimulation with the T cell-associated factors CD40L and IL-21, the non-conventional IgM^+^CD21^−^CD11R1^−^ B-1-like cell subpopulation demonstrated a higher potential to produce IgM that binds to *S. suis cps* 2 compared to non-conventional IgM^+^CD21^−^CD11R1^+^ and to conventional IgM^+^CD21^+^ cells (**Figure 4**). Taken together, conventional and non-conventional B cell subpopulations of eight-weeks-old pigs are able to secrete anti-*S. suis* IgM with highest levels produced by IgM^+^CD21^−^CD11R1^−^ B-1-like cells.

## 4 Discussion

*S. suis cps* 2 is one of the most important bacterial pathogens in piglet nurseries. IgM antibodies are known to be crucial for host defense against *S. suis* during the critical phase after weaning (15, 16, 30). The characteristic course of anti-*S. suis*-binding IgM antibodies crucial for bacterial clearance, published for different serotypes (*cps* 1, 7, 14/1) previously (30, 31), was confirmed for *cps* 2 in the present study: in the 4th week of life, levels of anti-*S. suis cps* 2 IgM are very low in porcine blood. In the following four weeks, there is an increase in IgM, which restricts bacterial growth *in vitro*. Furthermore, we show for the first time, that in pigs, the significant increase of *S. suis*-binding serum IgM between four and eight weeks of age is associated with a relative increase of peripheral non-conventional IgM^+^CD21^−^ B-1-like cells *in vivo*, particularly of the IgM^+^CD21 CD11R1^−^ subpopulation.

The two distinct subpopulations of porcine B-1-like cells, that differ with respect to the expression of CD11R1 (probably the porcine homolog of CD11b), have been described previously (24). In the present study the porcine IgM^+^CD21^−^CD11R1^−^ subpopulation, isolated from peripheral blood of 8-weeks-old pigs, was shown to constitutively secrete IgM, as described for human and murine innate B-1 cells (36, 37). Interestingly, despite the fact that comparable levels of total IgM were produced in response to TLR stimulation in porcine conventional and non-conventional B cell subpopulations, only TLR-induced IgM secreted by the two non-conventional IgM^+^CD21^−^ B-1-like cell subpopulations bound to *S. suis cps* 2. This highlights differences in reactivities of IgM antibodies secreted by TLR-activated B-1 versus B-2 cells likely due to distinct IgM BCR repertoires expressed by B-1 and B-2 cells. As observed in mouse B-1 cells (38), the concentrations of total IgM spontaneously secreted by porcine IgM^+^CD21^−^CD11R1^−^ B-1-like cells were very low, which might be the reason why *S. suis cps* 2-binding IgM was undetectable in their supernatants. Taken together, in contrast to conventional IgM^+^CD21^+^ B-2 cells, the IgM^+^CD21^−^ CD11R1^−^ B-1-like cell subpopulation constitutively secretes IgM and both non-conventional IgM^+^CD21^−^ B cell subpopulations (but not B-2 cells) produce anti-*S. suis* IgM upon TLR stimulation underlining their innate-like characteristics. Future experiments, including a detailed transcriptome and repertoire analysis will provide further insights into specific features of the two non-conventional B-1-like subpopulations in comparison to conventional B-2 cells. This can reveal whether porcine non-conventional B-1-like cells express a skewed variable heavy chain (VH) repertoire as shown for murine and human B-1 cells (39–41).

Interestingly, the increase in serum IgM between the fourth and eighth week of age without a corresponding rise in IgG indicates the absence of isotype class switch during this period. Stable IgM production without class switch is a characteristic feature of B-1 cells in mice (42). After experimental infection with *S. suis* there is an immediate increase in serum IgM, yet even 21 days post infection serum IgG does not increase significantly (43). In mice experimentally infected with *S. suis cps* 2, control of infection was mediated by *S. suis*-binding IgM and independent of germinal center reaction and of isotype switching. Nevertheless, production of anti-bacterial IgM antibodies was shown to be T cell-dependent, as T cell deficient mice could not control bacteremia, produce optimal anti-*S. suis* IgM titers, or elicit antibodies with opsonophagocytic activity (15). Interestingly, in light of this murine study indicating T cell dependent extrafollicular induction of anti-*S. suis* IgM, we observed that porcine B-1-like as well as B-2 cell subpopulations are capable of secreting *S. suis*-binding IgM upon stimulation with T cell-associated factors with highest levels in IgM^+^CD21^−^CD11R1^−^ B-1-like cells. Secreted IgM from all three subpopulations binds to *S. suis* and may therefore also play a role in bacterial killing *in vivo*.

Presently, we can only speculate on the specificity of the *S. suis cps* 2-binding IgM. A recent study indicates biological relevance of cross-reactivity of antibodies between the different *S. suis* serotypes *cps* 1 and *cps* 14 (31). Given that the capsule of *S. suis cps* 2 shares similarities with that of *cps* 1 and *cps* 14 (44), we hypothesize that the *S. suis cps* 2-binding IgM is not exclusively specific for *cps* 2 but may also bind to other *S. suis* serotypes. Noteworthy, the characteristic increase of serum IgM antibodies critical for bacterial clearance in pigs between four and eight weeks of age has not only been shown for different *S. suis* serotypes, but also for *Klebsiella pneumoniae* (45). Murine and human B-1 cells are the main producers of polyreactive IgM antibodies (20). It could be speculated that in pigs between the fourth and eighth week of life, B-1-like cells with innate features are also a major source of polyreactive IgM and that the antibacterial activity is not restricted to *S. suis* only but contributes to protection against different bacterial pathogens. However, this needs further investigation.

In conclusion, this is the first characterization of the porcine early IgM B cell response which identifies an increase of B-1-like cells in porcine peripheral blood between four and eight weeks of age concomitant with the increase of anti-*S. suis* IgM restricting bacterial growth *in vitro*. Our study provides the basis for future experiments which should reveal novel insights into the regulation of non-conventional and conventional B cell subpopulations in porcine early life host-pathogen interaction.

## Data Availability

The original contributions presented in the study are included in the article/**Supplementary Material**. Further inquiries can be directed to the corresponding author.
