# Supplementary material for: Porcine non-conventional B-1-like cells are a potent source of Streptococcus suis-binding IgM
Source: Front Immunol. 2024 Nov 18;15:1495359. doi: 10.3389/fimmu.2024.1495359 (PMC11609192; doi:10.3389/fimmu.2024.1495359)
Supplement: Supplementary file 1 [file Presentation1.pptx]

## Slide 1
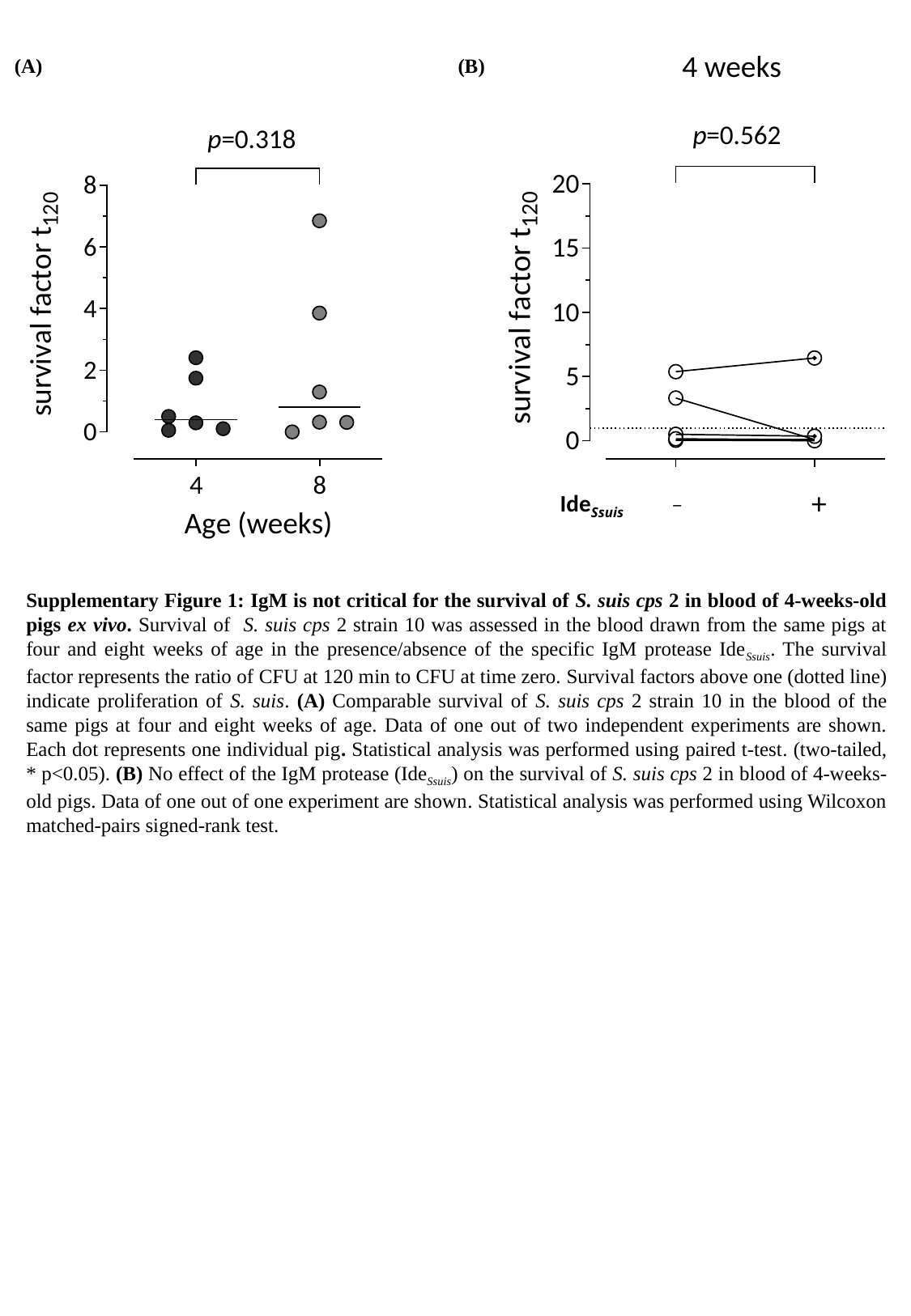

4 weeks
(A)
(B)
p=0.562
p=0.318
+
IdeSsuis
−
Supplementary Figure 1: IgM is not critical for the survival of S. suis cps 2 in blood of 4-weeks-old pigs ex vivo. Survival of S. suis cps 2 strain 10 was assessed in the blood drawn from the same pigs at four and eight weeks of age in the presence/absence of the specific IgM protease IdeSsuis. The survival factor represents the ratio of CFU at 120 min to CFU at time zero. Survival factors above one (dotted line) indicate proliferation of S. suis. (A) Comparable survival of S. suis cps 2 strain 10 in the blood of the same pigs at four and eight weeks of age. Data of one out of two independent experiments are shown. Each dot represents one individual pig. Statistical analysis was performed using paired t-test. (two-tailed, * p<0.05). (B) No effect of the IgM protease (IdeSsuis) on the survival of S. suis cps 2 in blood of 4-weeks-old pigs. Data of one out of one experiment are shown. Statistical analysis was performed using Wilcoxon matched-pairs signed-rank test.

## Slide 2
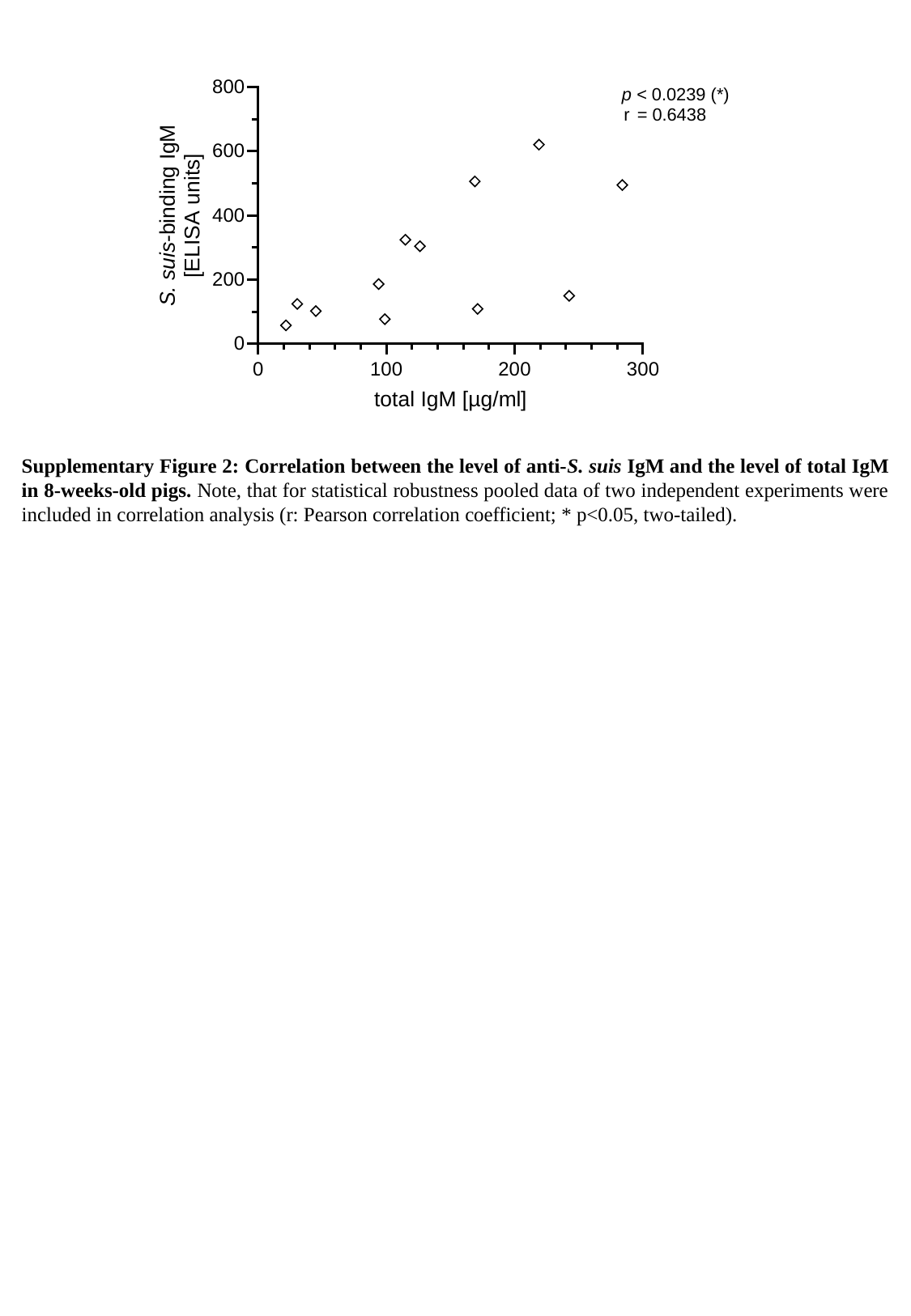

Supplementary Figure 2: Correlation between the level of anti-S. suis IgM and the level of total IgM in 8-weeks-old pigs. Note, that for statistical robustness pooled data of two independent experiments were included in correlation analysis (r: Pearson correlation coefficient; * p<0.05, two-tailed).
